# Costs and Savings Associated With Community Water Fluoridation Programs in Colorado

**Published:** 2005-10-15

**Authors:** Diane Brunson, Joan M O’Connell, Theresa Anselmo, Patrick W Sullivan

**Affiliations:** Colorado Department of Public Health and the Environment; University of Colorado at Denver and Health Science Center School of Medicine, Denver, Colo; Colorado Department of Public Health and the Environment, Denver, Colo; University of Colorado at Denver and Health Science Center School of Pharmacy, Denver, Colo

## Abstract

**Introduction:**

Local, state, and national health policy makers require information on the economic burden of oral disease and the cost-effectiveness of oral health programs to set policies and allocate resources. In this study, we estimate the cost savings associated with community water fluoridation programs (CWFPs) in Colorado and potential cost savings if Colorado communities without fluoridation programs or naturally high fluoride levels were to implement CWFPs.

**Methods:**

We developed an economic model to compare the costs associated with CWFPs with treatment savings achieved through averted tooth decay. Treatment savings included those associated with direct medical costs and indirect nonmedical costs (i.e., patient time spent on dental visit). We estimated program costs and treatment savings for each water system in Colorado in 2003 dollars. We obtained parameter estimates from published studies, national surveys, and other sources. We calculated net costs for Colorado water systems with existing CWFPs and potential net costs for systems without CWFPs. The analysis includes data for 172 public water systems in Colorado that serve populations of 1000 individuals or more. We used second-order Monte Carlo simulations to evaluate the inherent uncertainty of the model assumptions on the results and report the 95% credible range from the simulation model.

**Results:**

We estimated that Colorado CWFPs were associated with annual savings of $148.9 million (credible range, $115.1 million to $187.2 million) in 2003, or an average of $60.78 per person (credible range, $46.97 to $76.41). We estimated that Colorado would save an additional $46.6 million (credible range, $36.0 to $58.6 million) annually if CWFPs were implemented in the 52 water systems without such programs and for which fluoridation is recommended.

**Conclusion:**

Colorado realizes significant annual savings from CWFPs; additional savings and reductions in morbidity could be achieved if fluoridation programs were implemented in other areas.

## Introduction

In 2000, the U.S. Department of Health and Human Services released the first national oral health report, *Oral Health in America: A Report of the Surgeon General* (1), which described a "'silent epidemic' of dental and oral diseases." Compared with other health conditions such as diabetes and depression (2,3), less is known about spending for oral disease in the United States because many spending estimates include only services provided in dental offices (4-7; A. Martin, written communication, March 2005). According to 2003 estimates (4,5, A. Martin, written communication, March 2005), spending for services provided in dental offices averaged $306 per capita in Colorado, with total annual spending for these services in Colorado estimated to be $1.3 billion. These estimates do not include dental services provided in other settings, such as hospitals, nor do they include services for other oral health conditions, such as oral cancer. Furthermore, the amount spent on oral disease may surpass the amount spent on medical services (both dental and other services) to treat such disease because of costs related to adverse health effects, productivity losses, and reduced quality of life.

It is important for health policy makers, health education specialists, health care providers, and the news media to have state-specific quantitative information on the impact of oral disease prevention strategies to maintain support for existing programs and promote implementation of new programs. Because of limited information on the economic burden associated with oral disease, the state of Colorado initiated a process to quantify the burden by building on data compiled for the state's Oral Health Surveillance System. The goal of the Oral Health Economic Burden Model is to quantify short-term and long-term medical and nonmedical costs associated with poor oral health to assist Colorado state and local policy makers in designing policies and optimizing allocation of health resources to improve oral health. The purpose of this article is to describe one component of the Oral Health Economic Burden Model; the component was used to estimate costs and savings associated with community water fluoridation programs (CWFPs).

Community water fluoridation is defined as the adjustment of fluoride levels in public drinking water systems for the prevention of dental decay; it has been shown to be one of the most cost-effective public health strategies in the United States (8) and is recognized as one of the 10 great public health achievements of the 20th century (9). For most communities with CWFPs, the adjustment of fluoride levels requires the addition of fluoride compounds to increase the fluoride level to the recommended level; for a small percentage of communities, the adjustment requires the addition of water with lower concentrations of fluoride compounds to decrease the fluoride level to the recommended level.

The nonfederal, independent Task Force on Community Preventive Services (Task Force) completed a systematic review of the evidence of effectiveness for CWFPs (8). Findings indicated a 29.1% median decrease in dental caries among children aged 4 to 17 years in communities with CWFPs. This finding led the Task Force to strongly recommend that CWFPs be included as part of a comprehensive population-based strategy to prevent or control dental caries in communities. The systematic review by the Task Force on the cost-effectiveness of CWFPs found that among the five studies with sufficient data, CWFPs resulted in cost savings, with the savings in dental treatment costs exceeding fluoridation program costs for systems servicing populations of 20,000 or more (8).

In 2001, Griffin et al conducted the most comprehensive data-driven economic evaluation of community water fluoridation since the 1980s and reported on the net costs (program costs minus treatment savings) of CWFPs by community size (10). We adapted this model for use at the state level to estimate the net costs associated with existing CWFPs in Colorado and the potential net costs if communities without CWFPs, and for which such a program is recommended, were to implement fluoridation programs.

In 2005, Colorado met the *Healthy People 2010* objective (21-9) of 75% or more of people using optimally fluoridated water through community water systems (11,12). The actual percentage in Colorado, however, was just above 75%. Because communities with CWFPs face challenges in retaining water fluoridation programs, and communities without programs require information to make implementation decisions, it is important that data on CWFP costs and treatment savings be available at the state level.

## Methods

Annual CWFP net costs in Colorado were estimated by comparing annual fluoridation program costs with treatment savings associated with averted tooth decay, where


*(1) Net Costs_water system_ = Program Costs_water system_*
*- Treatment Savings_water system._*

We assumed that the fluoride level of the water system was adjusted to the Centers for Disease Control and Prevention's (CDC's) recommended fluoride concentration level, based on the average temperature and altitude of the community. These levels range from 0.7 ppm to 1.2 ppm (13). If the difference between the CDC-recommended level and the natural fluoride level is 0.3 ppm or greater for a water system, the CDC recommends the implementation of a CWFP (K. Duchon, PhD, written communication, January 2005). For example, if the CDC-recommended fluoride level for a water system was 1.0 ppm and the naturally occurring level was 0.4 ppm (a difference of 0.6 ppm), the water system was included in our list of systems for which fluoride was recommended.

Our analysis included data from the Water Fluoride Reporting System for 172 public water systems in Colorado that served populations of 1000 or more in 2004 (11). The water systems include 61 water systems with CWFPs and 111 systems without CWFPs. Among the 111 systems without programs, CWFPs were recommended for 52, based on CDC recommendations. Among these systems, 32 systems had naturally occurring fluoride levels of less than 0.3 ppm, 9 had levels between 0.3 ppm and 0.5 ppm, and 11 had levels of more than 0.5 ppm. The remaining 59 systems had naturally occurring fluoride levels lower than the CDC-recommended level (yet within the 0.3 cutoff) or had levels equal to or greater than the recommended level. CWFPs were not recommended for these 59 water systems; we refer to these systems as having naturally high fluoride levels. Information on the size of populations served, according to the fluoride status of the water system, is provided in Table 1.

Our analysis adhered to the recommendations of the Panel on Cost-Effectiveness in Health and Medicine (14,15). We reference the work of Griffin et al (10) in describing the methods we used to estimate CWFP net costs, noting modifications. When possible, we used state and local data sources such as the Water Fluoridation Reporting System (11) for information on fluoride levels of local water systems and Colorado Vital Statistics for population and mortality data (4). Other data sources included regional and national data, published studies, and expert opinion.

CWFP costs and treatment savings were estimated from a societal perspective, with costs and savings provided in 2003 dollars using a discount rate of 3%. The benefit from water fluoridation is primarily topical; fluoridation prevents decay in teeth after they have erupted (16). As such, we estimated program treatment savings for individuals aged 5 years and older and included costs for permanent teeth only.

### Costs associated with CWFPs

CWFP cost estimates were based on data reported in a published study that included both one-time fixed costs and annual operating costs for communities in Florida that ranged in population from fewer than 5000 to more than 400,000 (17). These costs are the most complete costs reported in the literature. Even though these data are for the late 1980s, fluoridation technology has not changed in a way that would limit the usefulness of these data in our analysis.

We used data for 42 systems that fluoridated water with hydrofluosilicic acid, which is the most commonly used fluoridation compound. One-time fixed costs included general equipment, testing and safety equipment, installation, and engineering consultant fees. These costs were depreciated over a 15-year period with no salvage value, using a 3% discount rate. The annual operating costs included fluoride compounds, labor, maintenance, and accessory supplies. These annual costs were adjusted for inflation to 2003. The Water, Sewage, and Maintenance cost component of the Consumer Price Index (18) was used to adjust chemical and labor costs. The *Engineering News-Record* Building Cost Index (19) was used to adjust capital costs. Operating and annual capital costs in 2003 dollars were summed to obtain total program costs and to calculate an annual mean CWFP per-person cost by water system size (Table 2).

We estimated annual CWFP costs for each water system as follows:


*(2) Program Costs_water system_ = Population_water system_*
*× Program Cost Per Person_size of water system._*

### Treatment savings associated with CWFPs

Annual treatment savings depend on both the averted decay attributable to CWFPs and the costs of treatment over the lifetime of the tooth that would have occurred without CWFPs:


*(3) Treatment Savings_per person_ = Averted Decay_per person_*
×* Lifetime-Treatment Cost_per person._*

**1. Estimating annual averted decay attributable to CWFPs**

Averted decay is the product of the percentage reduction in tooth decay associated with CWFP (program effectiveness) and the annual per person decay increment in nonfluoridated areas:


*(4) Averted Decay_per person_ = CWFP Effectiveness × Decay Increment in Nonfluoridated Areas_per person._*

Estimated age-specific annual decay increments (the number of new decayed tooth surfaces per year) for nonfluoridated communities were obtained from two sources. The decay increment in nonfluoridated areas for individuals aged younger than 45 years was derived by Griffin et al (10) from two national studies (20,21) that were conducted between 1985 and 1987 and that included information on community water fluoridation status. One study was of U.S. schoolchildren; the other study was of employed adults and seniors. The researchers estimated the annual decay increment (including root surfaces) to be 0.77 surfaces for individuals aged 6 to 17 years and 1.09 surfaces for individuals 18 to 44 years. Given the decline in decay increment since 1980 (22), we adjusted the annual decay increment for a secular trend (20.9%) based on an analysis of data from the mid-1980s and a more recent survey (23). However, the decay increment for individuals aged 45 to 64 years in nonfluoridated areas was somewhat low, and no estimate was provided for individuals aged 65 and older. Consequently, we used findings from a recent meta-analysis of 11 studies conducted between 1983 and 1999 for individuals with and without exposure to fluoride to estimate the annual decay increment for individuals 45 years and older in nonfluoridated areas (24). In 2004, Griffin et al estimated the total (coronal and root) decay increment for individuals 45 years and older to be 1.31; we used this estimate for individuals aged 65 and older. Because of lower rates of root decay among individuals aged 45 to 64 years compared with individuals aged 65 years and older (24,25), we used an estimated total decay increment of 1.08 for individuals aged 45 to 64 years (S. Griffin, PhD, oral communication, June 2005). We did not adjust the more recent estimates for a secular trend; if the decay increment declined recently because of improvements in oral health, use of these estimates may positively bias results. On the other hand, use of a decay increment based on data for individuals with and without exposure to fluoride as estimates for nonfluoridated increments and exclusion of avoided caries in the primary dentition (i.e., baby teeth) from the model may negatively bias results. It was difficult to assess the directional impact of using these four age-specific estimates on CWFP treatment savings.

We assumed that the population distribution of each water system was similar to the state's total population and used the age-specific rates and the 2003 age distribution in Colorado (4) for individuals aged 5 years and older to derive an annual age-adjusted decay increment for Colorado nonfluoridated communities (0.78 surfaces per year per person) (Table 3). In addition, the age-specific rates were used to estimate the lifetime-treatment cost of applying and maintaining a restoration.

Based on the findings of studies published in the 1990s (26-28) and on national survey data (20), Griffin et al in 2001 estimated that CWFPs reduced the decay increment by approximately 25%. This estimate of CWFP effectiveness is lower than earlier estimates because fluoride is now available from multiple sources (e.g., toothpaste, mouth rinses, professional applications, foods and beverages produced in areas with CWFPs) in addition to local drinking water (22). We multiplied the estimated annual decay increment for nonfluoridated communities (0.78 surfaces) by the percentage of reduction (25%) estimated by Griffin et al to obtain the averted annual per-person decay increment attributable to CWFPs. This value, equal to 0.20 surfaces, was multiplied by the size of the population exposed to CWFPs to yield the total number of decayed surfaces averted due to 1 year of exposure to water fluoridation.

**2. Lifetime cost of treatment: applying and maintaining a restoration**

A restoration requires maintenance over the time the tooth remains in the mouth. We derived the discounted lifetime cost of applying and maintaining a restoration using the approach employed by Griffin et al (10) with noted modifications. For each age group, we estimated the discounted lifetime-treatment cost from 1) the number of initial restorations averted because of fluoridation, 2) the number of averted replacement restorations expected over the course of a lifetime, 3) the types of restorations used for initial and replacement procedures, and 4) the costs of the associated dental visits. We combined the age-specific results with data on age distribution in Colorado to estimate an age-adjusted lifetime-treatment cost of applying and maintaining a restoration.

For each age group, the first step in estimating the lifetime-treatment cost was to derive the expected number of initial restorations, which we assumed to be the number of decayed surfaces averted because of 1 year of exposure to water fluoridation. We estimated the number of replacement restorations by using the midpoint of each age group listed in Table 3 and the expected life of the restorations. Based on published studies (29-33), Griffin et al assumed that the expected life of a single amalgam restoration was 12 years (10). We used this value, and we assumed that multisurface amalgam and composite restorations have a similar expected life; the expected life of a crown was assumed to be 24 years. Consequently, an adolescent who has an initial restoration at age 12 may have three to four replacement restorations; a person who has an initial restoration at age 60 may have only one. For each age group, we estimated the total number of replacement restorations, given the mortality rate (4), the probability of having the tooth (25), and the probability of a tooth extraction resulting from tooth decay rather than other reasons (34).

Next, we derived the cost of initial and replacement restorations using information on the types of materials used and the number of surfaces restored. The frequency of restoration procedures was obtained from age-specific restoration information calculated from private-sector administrative-claims data for 2002 from the largest dental insurer in Colorado (J.M.O., unpublished data, 2004). We recognized that privately insured individuals may obtain a different mix of services than that obtained by individuals without such coverage (7). For this analysis, we assumed services provided to individuals with private coverage represent practice standards and consumer expectations. We used these data as the best indicators of the value of maintaining a tooth; the data account for the long-term value of a tooth, including nutritional, other health, and quality-of-life considerations that are not quantified but well-recognized (1).

We used data for five groups of restorations: single-surface amalgam, two-or-more-surfaces amalgam, single-surface composite, two-or-more-surfaces composite, and crowns. Over a lifetime, a restoration is often replaced with many restorations, resulting in an increased number of restored surfaces (35,36). For this reason, we used age as a proxy for the types of restorations used for initial and replacement restorations. The distribution of initial restorations was assumed to be similar to restorations for individuals aged 6 to 17 years, excluding crowns. Crowns were excluded because most crowns for this age group may be associated with accidents rather than caries. Accordingly, 38% of initial restorations were assumed to be single-surface amalgam, 23% were two-or-more-surfaces amalgam, 24% were single-surface composite, and 15% were two-or-more-surfaces composite restorations.

Likewise, restorations for individuals aged 18 to 29 years were assumed to be similar to the distribution for first-replacement restorations; restorations for individuals aged 30 to 41 were assumed to be similar to the distribution for second-replacement restorations; restorations for individuals aged 42 to 53 were assumed to be similar to the distribution for third-replacement restorations; and restorations for individuals aged 54 to 64 were assumed to be similar to the distribution for fourth-replacement restorations. To control for the use of crowns for purposes other than decay, we assumed that the rate of such usage in older age groups would be similar to the rate for individuals aged 6 to 17 years; we adjusted the use of crowns for the older age groups accordingly. As such, second-replacement restorations were assumed to include 20% single-surface amalgam, 27% two-or-more-surfaces amalgam, 18% single-surface composite, 21% two-or-more-surfaces composite, and 13% crowns. This approach may be conservative because restorations for individuals at older ages include initial restorations as well as replacement restorations. Information on root canal treatments, bridges, and other restorative procedures were not included in our restoration calculations.

We assumed dental fees approximated the cost of resources used to provide dental services, and we used the reported fees for amalgam restorations, composite restorations, five of the most frequently used crowns, and extractions from the *2003 Survey of Dental Fees* (37) for the Mountain Region, which includes Colorado, for procedure cost estimates. We estimated the average cost of initial and first-through-fourth replacement restorations using the reported fees and distribution of restoration procedures by age group (Table 4).

The cost of each dental visit included direct medical costs for the restoration and the nonmedical costs associated with patient time spent for the dental visit, where 


*(5) Dental Visit Cost_per visit_ = Direct Medical Cost for Restoration_per visit_ + Patient Time Cost_per visit._*

The time spent receiving dental care and traveling to the dental office was estimated to be 1.6 hours per visit, based on published data on travel time, office-visit wait time, and actual treatment time in dental offices (38). The cost of a patient's time was quantified using a national estimate for the value of 1 hour of activity for men and women in 2000 (11), updated to 2003 dollars ($20.11) (39). This estimate was used to value time for all individuals, including individuals employed both inside and outside of the home.

For each age group, we used estimates of the number of dental visits and related costs to calculate a discounted lifetime-treatment cost of applying and maintaining a restoration. For example, for an age group with three potential replacement restorations, the per-person discounted lifetime cost of applying and maintaining a restoration was calculated by using the following formula:


*(6) Lifetime-Treatment Cost_per person_ =_ _ (CR_initial_/D) + ([{P_tooth _ × CR_replace1_} + {P_extract _ × CE}]/D) + ([{P_tooth _ × CR_replace2_} + {P_extract _ × CE}]/D) + ([{P_tooth _ × CR_replace3_} + {P_extract _ × CE}]/D)*

where *CR* is the cost of restoration (including an initial dental visit [*CR_initial_*] and three replacement dental visits [*CR_replace1_*, *CR_replace2_*, and *CR_replace3_*]); *D* is the discount rate for the time period;* P_tooth_* is the probability that the tooth exists; *P_extract_*  is the probability that the tooth will be extracted because of decay; and *CE* is the cost of a visit for an extraction. Using the per-person lifetime-treatment cost for each age group and the 2003 age distribution, we estimated the age-adjusted per-person discounted lifetime-treatment cost to be $290.27. We multiplied this value by the estimated per-person annual averted decay increment attributable to fluoridation (0.20 surfaces) and arrived at a per-person annual treatment savings of $58.05.

Similar to Griffin et al (10), we assumed that the adverse effects of exposure to water fluoridation were negligible (40) and did not adjust CWFP savings for such effects. CWFP annual treatment savings for a water system were estimated by multiplying the water system population by the per-person annual treatment savings:


*(7) Treatment Savings_water system_ =  Population_water system _ × Treatment Savings_per person._*

### Analysis

We first estimated total CWFP net costs for the existing 61 CWFPs in Colorado. Second, we used the same methodology to estimate total net program costs potentially associated with implementing CWFPs in 52 water systems recommended for fluoridation. The total CWFP net program costs represent the sum of net costs for each water system included in each estimate.

We conducted sensitivity analyses to evaluate the inherent uncertainty of assumptions for the input variables on the model results. First, we employed univariate sensitivity analyses. Then we used second-order Monte Carlo probabilistic sensitivity analyses that allowed CWFP costs and effectiveness, decay increment, dental fees, and patient-time costs to vary simultaneously. The 10,000 Monte Carlo simulations were conducted using TreeAge Pro 2005 (TreeAge Software, Inc, Williamstown, Mass). The TreeAge Pro model was linked to a Microsoft Excel spreadsheet to estimate water-system–specific program costs and treatment savings. The Monte Carlo simulation is referred to as probabilistic sensitivity analysis because each input-parameter estimate that was not a fixed value was assigned a probability distribution that reflected beliefs about the feasible range of mean values. For each simulation, a value from each probability distribution was drawn for each parameter simultaneously. The CWFP costs and treatment savings were then calculated for each water system using these values as the input parameters. The simulation repeated this process 10,000 times to produce a range of possible values. We report the absolute value of CWFP net costs baseline estimates with a 95% credible range (the 2.5th to 97.5th percentiles of the 10,000 simulations) from the simulation model.

Probability distributions were based on what was known about the parameter estimates: the age-specific decay rate for nonfluoridated areas, the number of hours associated with a dental visit, and the dollar value of 1 hour of time were assumed to have normal distributions. The fluoride program effectiveness rate and the secular trend for the decay rate in nonfluoridated areas were represented as a β distribution because they were expected to be normally distributed but restricted to values between 0 and 1. The CWFP program costs and restoration costs were characterized as γ distributions to ensure positive values.

In addition, we estimated net costs associated with CWFP implementation in the 52 water systems currently without fluoridation programs, using two alternative model specifications. In one model, we excluded from the analysis two water systems with populations greater than 90,000 and average natural fluoride levels of 0.6 ppm to 0.7 ppm. The difference between the CDC-recommended fluoride level and the natural fluoride level for the two systems was only slightly higher than 0.3 ppm. In the second model, we adjusted CWFP effectiveness by the natural fluoride level in the local communities using a linear model (22,41). We used the estimated effectiveness of a 25% decrease in decay for water systems with natural fluoride levels of 0.3 ppm or less. For systems with fluoride levels of 0.31 to 0.39 ppm, we used an effectiveness rate of 23%; for levels of 0.40 to 0.49 ppm, a rate of 19%; for levels of 0.50 to 0.59 ppm, a rate of 15%; and for levels of 0.60 to 0.69 ppm, a rate of 10%.

## Results

Existing CWFPs in Colorado were associated with negative net annual costs (hereon referred to as *net savings*) of $148.9 million (credible range [CR], $115.1–$187.2 million) in 2003 or an average of $60.78 per person (CR, $46.97–$76.41). When presented as a ratio of savings (benefits) to costs, the estimates ranged from $21.82 for small water systems to $135.00 for large systems. We varied the parameter estimates for the decay increments, program effectiveness, and program cost by ±15% from the baseline value to assess which parameter estimates had the greatest impact on program net savings (Figure). The results of the sensitivity analyses indicated that CWFP net savings were most sensitive to changes in the baseline estimates for CWFP effectiveness, as measured by the percentage of reduction in the decay increment and the decay increment in nonfluoridated areas for individuals aged 18 to 44 years.

**Figure:**
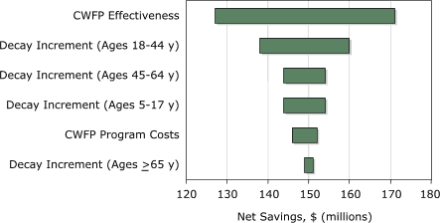
Univariate sensitivity analysis of the variation in model parameter estimates on net savings in dental care costs resulting from community water fluoridation programs (CWFPs) in 61 water systems in Colorado. Model inputs were varied by ±15% from the baseline value to assess parameter estimates with the greatest impact on the variation in CWFP net savings.

**Table:** 

Using the baseline assumptions, we estimated that Colorado would save an additional $46.6 million (CR, $36.0–$58.6 million) annually if CWFPs were implemented in the 52 nonfluoridated water systems for which fluoridation is recommended. Approximately 80% of these savings would be realized for the six large water systems that serve populations greater than 20,000. However, two of the six water systems serve more than 90,000 individuals, and the difference between the CDC-recommended fluoride level and natural level was slightly more than 0.3 ppm. When these two systems were excluded from the analysis, potential savings in the other 50 water systems were estimated to total $34.4 million. We conducted one variation of the model by adjusting the CWFP effect on reducing decay for the presence of natural fluoride levels. Using lower rates of fluoride effectiveness for areas with fluoride levels greater than 0.3 ppm, net savings were estimated to be $39.0 million annually.

## Discussion

Although Colorado realizes significant annual savings from existing CWFPs, additional savings and reductions in morbidity could be achieved if fluoridation programs were implemented in other areas. Approximately 80% of the additional savings would be realized if six large water systems that serve populations greater than 20,000 implemented fluoridation programs.

There are limitations to our model and its assumptions that affect these estimates. First, CWFPs use different types of fluoride compounds. We based our model on the estimated cost of using hydrofluosilicic acid; we selected this compound because it is the most widely used fluoridation compound (11) and thought to be the most economical (17). Second, the fluoridation program cost estimates represent average costs by program size and include repairs and maintenance. These cost estimates, however, may not represent the actual costs for a particular water system during any one period. Third, the model includes assumptions on decay increment, fluoride effectiveness, and use of restorations and extractions based on cross-sectional data. We were not able to identify data sources with longitudinal information. We used more than one data source to estimate the decay increment for the four age groups; we previously noted limitations of their use. The decay-increment estimates for individuals aged 45 to 64 years and 65 years and older were based on data for individuals with and without access to fluoride. Use of these estimates and exclusion of avoided caries in the primary dentition from the CWFP treatment savings negatively biased the results.

A fourth limitation concerns the effectiveness of CWFPs at reducing the decay increment. The effectiveness of existing CWFPs may be underestimated because individuals living in nonfluoridated areas benefit from the diffusion of fluoride into their communities through foods and beverages produced in fluoridated areas; the effectiveness of new CWFPs may be overestimated because of diffusion. Furthermore, fluoride is now available from multiple sources such as toothpaste, mouth rinses, and professional applications; savings associated with CWFPs are reduced as use of these other fluoride sources increases. CWFP savings would also be reduced if recommended fluoride levels were lower. For example, the World Health Organization recommends a range of 0.5 ppm to 1.0 ppm; this range recognizes that variation in diet, temperature, culture, and exposure to other sources of fluoride must be taken into account (42).

Fifth, the model accounts for time spent obtaining dental care, but the model does not account for the loss in productivity due to morbidity. The inclusion of productivity losses would have increased CWFP treatment savings. Sixth, the model estimates the value of treating a tooth with decay using 1) patterns of use of dental services among individuals with private-sector dental coverage and 2) dental fees that assume competitive markets. Patterns of use of dental services among individuals without private coverage differ from individuals with such coverage; we assumed that private-sector patterns of use reflect the long-term value of maintaining a tooth for quality-of-life, nutritional, or other health considerations. We did not adjust for differences by insurance coverage or income level. Finally, the model included age-specific rates for estimates of dental-procedure use and for the probability of a tooth extraction. We did not include variability for other estimates because of the complexity of using age-specific rates for these two estimates.

When possible, we used conservative assumptions in the model to negatively bias the net-cost estimates of CWFPs. However, as noted previously, it is difficult to assess the directional impact of other assumptions, and some may positively bias results. Health economic models are not designed to perfectly reflect all of the complexities of the real world (43). Given the limitations discussed, we believe this model, which accounts for some degree of uncertainty, provides useful information about the costs and savings associated with CWFPs. As new data and information become available, this model may be updated.

Traditional messages on fluoridation have been, "it prevents caries," "it saves money," and "it's cost-effective." The model used in this analysis provides Colorado-specific estimates of CWFP savings and may be replicated for other states. Such information may be used by public health practitioners and policy makers at all levels to promote continued support for existing CWFPs and implementation of new programs.

This study documents net costs of CWFPs for water systems serving populations of more than 1000. In addition to information on the costs and savings associated with CWFPs, it is important for communities to have information on decay increment and on all fluoride sources to be able to thoroughly evaluate the costs and benefits of CWFPs. It is also important to assess costs and savings of CWFPs and other fluoride delivery solutions, such as fluoride varnish, mouth rinse, and tablets, for populations in smaller communities. Finally, statewide cost estimates for other oral health conditions and savings associated with other oral health programs are needed to further inform state policy and spending decisions to reduce rates of oral disease in Colorado.

## Figures and Tables

**Table 1:** Public Water Systems by Population Size and Status of Community Water Fluoridation Program (CWFP), Colorado, 2004^a^

**Size of Population Served by Water System During 2004**	**Water Systems With CWFPs**		**Water Systems for Which CWFPs Are Recommended**		**Water Systems With Naturally High Fluoride Levels**	

	**No. Systems**	**Total Population Served by Water System**	**No. Systems**	**Total Population Served by Water System**	**No. Systems**	**Total Population Served by Water Systems**
≥100,000	6	1,704,765	2	430,250	0	0
50,000-99,999	4	290,996	1	90,700	0	0
20,000-49,999	6	183,929	3	91,357	5	174,968
10,000-19,999	10	142,850	2	20,308	12	181,211
5000-9999	7	54,376	10	70,370	8	56,723
1000-4999	28	72,758	34	66,302	34	80,650
**Total**	**61**	**2,449,674**	**52**	**769,287**	**59**	**493,552**

a Source: Colorado Department of Public Health and Environment (11).

**Table 2:** Estimated Mean Annual Cost per Person for Community Water Fluoridation Program by Size of Population Served by Water System, Colorado, 2004^a^

**Size of Population Served by Water System**	**Estimated Mean Annual Cost per Person, $**	**SE, $**
1000-4999	2.66	0.40
5000-9999	1.44	0.09
10,000-19,999	0.93	0.09
≥20,000	0.43	0.05

a Sources: Ringelberg ML, Allen SJ, Brown LJ (17); U.S. Department of Labor, Bureau of Labor Statistics (18); Engineering News-Record (19).

**Table 3:** Estimates of Annual Decay Increment by Age and the Age-adjusted Decay Increment in Nonfluoridated Areas, Colorado, 2004

**Age, y**	**2003 Colorado Population Age Distribution, 5 Years and OlderNo. (%)^a^**	**Estimate of U.S. Average Annual Decay Increment in Nonfluoridated Areas**	**Estimate of U.S. Average Annual Decay Increment in Nonfluoridated Areas, Adjusted for Secular Trend^b^**	**Colorado Age-adjusted Estimate of Annual Decay Increment in Nonfluoridated Areas^c^**
5-17	836,770 (20.0)	0.77^d^	0.61	0.12
18-44	1,871,371 (44.7)	1.09^d^	0.86	0.38
45-64	1,054,858 (25.2)	1.08^e^	1.08	0.27
≥65	428,027 (10.2)	1.31^e^	1.31	0.13
≥5	4,191,026 (100.0)	NC^f^	NC^f^	0.78

a Source: Colorado Department of Public Health and Environment (4).

b The decay increments for ages 5 to 17 years and 18 to 44 years were adjusted for decay increment decreases that occurred since 1980 (22,23).

c The values in this column were calculated for each age group (5-5-17 years, 18-44 years, 45-64 years, and ≥65 years) by multiplying the percentage value in the first column (2003 Colorado Population Age Distribution) and the value in the third column (Estimate of U.S. Average Annual Decay Increment in Nonfluoridated Areas, Adjusted for Secular Trend).

d Source: Griffin et al (10).

e Sources: Griffin et al (24); S. Griffin, oral communication, June 2005.

f NC indicates not calculated.

**Table 4:** Dental Procedure Restoration Fees and Estimated Initial and Replacement Costs for Restorations, United States, 2003

**Direct Medical Costs (Procedures)^a^**	**Mean, $**	**SE, $**
D2140 Amalgam, 1 surface, primary or permanent	85	2.3
D2150 Amalgam, 2 surfaces, primary or permanent	108	3.2
D2160 Amalgam, 3 surfaces, primary or permanent	129	4.1
D2161 Amalgam, 4 or more surfaces, primary or permanent	154	4.4
D2330 Resin-based composite, 1 surface, anterior	105	2.5
D2331 Resin-based composite, 2 surfaces, anterior	132	3.3
D2332 Resin-based composite, 3 surfaces, anterior	162	4.0
D2335 Resin-based composite, 4 or more surfaces or involving incisal angle, anterior	200	5.3
D2391 Resin-based composite, 1 surface, posterior	113	2.6
D2392 Resin-based composite, 2 surfaces, posterior	150	3.6
D2393 Resin-based composite, 3 surfaces, posterior	185	4.6
D2394 Resin-based composite, 4 or more surfaces, posterior	210	6.3
D2720 Crown, resin with high noble metal	708	28.2
D2750 Crown, porcelain fused to high noble metal	742	10.7
D2751 Crown, porcelain fused to predominantly based metal	684	11.9
D2752 Crown, porcelain fused to noble metal	714	9.9
D2790 Crown, full cast high noble metal	742	121.8
D7140 Extraction, erupted tooth or exposed root	96	2.0

**Indirect Costs (Time Spent Obtaining Dental Care)**	**Mean**	**SE^b^**

No. hours spent obtaining dental treatment^c^	1.6	0.16
Value of 1 hour of time^d^, $	20.11	2.01

**Total Costs (Direct Medical Costs + Indirect Costs)^e^**	**Mean Estimate, $**	**SE^b^, $**

Initial restoration	141	14.1
First replacement restoration	174	17.4
Second replacement restoration	229	22.9
Third replacement restoration	286	28.6
Fourth replacement restoration	304	30.4
Extraction	128	12.8

a Source: American Dental Association (37).

b Standard errors were estimated to be 10% of the baseline estimate.

c Source: American Dental Association (38).

d Sources: Haddix et al (15); U.S. Department of Labor, Bureau of Labor Statistics (39).

e The medical costs for the initial and first-replacement through fourth-replacement restoration costs were estimated using the reported dental procedure fees (37) and the age-specific distribution of restoration procedure types (J.M.O., unpublished data, 2004).
